# Chemical carcinogenesis – mode of action to inform quantitative human risk

**DOI:** 10.1186/1753-6561-7-S2-K10

**Published:** 2013-04-04

**Authors:** João Lauro V de Camargo

**Affiliations:** 1 Department of Pathology – Faculty of Medicine, São Paulo State University, UNESP, Botucatu, São Paulo, Brazil

## 

The progressive public and governmental awareness that we live in a chemical world, where both natural and man-made substances impose specific risks to living organisms, raised the need for regulatory and interventional measures to protect human health. Naturally, regulatory measures depend on identification of the hazard and the risk imposed by suspected chemicals. In the absence of human data, laboratory models have been used to identify target organs, adverse effects, and critical toxicological doses, all of which contribute to subsidize specific regulations. A case study is presented to illustrate how basic research can contribute to regulatory measures aiming to control human cancer risk due to environmental contaminants.

Diuron (3-(3,4-dichlorophenyl)-1,1-dimethylurea) is a substituted urea herbicide acting through inhibition of plant photosynthesis. In a 2-yr long experiment, Wistar rats fed diuron at 2,500 ppm (about 157 mg/kg.day for both sexes) developed urothelial carcinomas in the urinary bladder. The non-observed effect level (NOEL) for carcinogenicity was 25 ppm (1.35 mg/kg.day), based on urothelial hyperplasia and tumors [1]. Accordingly, the U.S. Environmental Protection Agency evaluated diuron as a “known/likely carcinogen to humans” [1,2]. Despite this classification, the quantitative risk associated to diuron was considered of no concern [2] and the herbicide is worldwide applied on main agricultural crops such as soy, cotton and sugar cane, among other uses. The impact of chronic low-dose exposure to diuron on human health has not been determined.

The standard procedure for identifying chemical carcinogens is the long-term bioassay with rodents. Although presenting a 40-year long history of usefulness, this 2-year assay bears important limitations, such as high cost and operational complexity, and also because it was not designed to provide information about the toxicological mode of action (MoA) of chemicals. As for any experiment with laboratory animals, extrapolating the results to the human situation is not a simple task, and may compromise human risk assessment. To inform risk assessment, a framework was proposed for systematic and detailed evaluation of the MoA in laboratory animals and for evaluating the human relevance of experimental results [3-5].

The genotoxic/mutagenic potentials of diuron are accepted to be negative [1,6,7]. Therefore, the carcinogenic MoA of diuron in the rat urinary bladder should be predominantly non-genotoxic. In an early study, rats fed diuron at 2,500 ppm during 20 weeks developed urothelial necrosis – observed ultrastructurally -, regenerative cell proliferation and urothelial hyperplasia [6]. To test the hypothesis that urinary crystals and amorphous precipitates could act as microabrasives to the mucosa causing cell death and regenerative hyperplasia that lead to urothelial carcinogenesis, rats were fed 2,500 ppm of diuron either with or without NH_4_Cl 10,000 to acidify urine during 15 to 30 weeks [7]. Groups fed diuron+NH_4_ showed reduced amount of urinary solids in the urine; nevertheless, urothelial necrosis and simple hyperplasia (SH), a stochastic preneoplastic urothelial lesion, were observed both in diuron- and diuron+NH_4_Cl treated animals. Therefore, chemically induced cytotoxicity and not abrasion by urinary solids seems to be the initial key event for diuron-induced rat urothelial carcinogenesis. These urothelial lesions start at the very 1^st^ day of exposure to 2,500 ppm and encompass cell swelling followed by necrosis, exfoliation and piling up of cells indicative of hyperplasia [8]. A recent study indicated that 500 ppm can also induce urothelial citotoxicity [9].

Regarding dose-response and the molecular pathways involved, rats were fed diuron at different concentrations from 60 ppm up to 2,500 ppm for 7 days and 20 weeks. After 7 days no significant urothelial lesions were observed by light or electron microscopy but gene expression profile (Affymetrix microarrays) indicated altered pathways related to cell-to-cell interactions in a dose-response manner, suggesting that the early influence of diuron and/or its metabolites on the urothelium may result in loss of cell-to-cell adhesion and local tissue disorganization [10]. By the week 20^th^, rats fed 1,250 or 2,500 ppm, but not those fed 125 ppm or lower, developed SH [11]. Accordingly, gene expression data suggested that low levels of diuron induced pathways involved in the maintenance of cellular homeostasis while higher levels were associated to increased cell metabolism, oxidative stress and cell death followed by sustained hyperplasia. Although based on preneoplastic alterations, these data allow indicating diuron at 1,250 ppm as potentially carcinogenic as 2,500 ppm and assuming 125 ppm as a no effect level (NOEL) for urothelial alterations [11].

This case study illustrates how translational non-clinical research can help to understand and eventually control environmentally-related human cancer hazards. Optical and ultrastructural microscopies, genotoxicity assays, cell proliferation assays, and genome-wide transcriptional profiling disclosed key events of diuron-induced rat urothelial cytotoxicity and potential carcinogenesis. They also indicated that a threshold dose may exist below which no carcinogenic process is launched. Data still missing to support sound quantitative human risk assessment refers to the evaluation of human relevance of the key events verified in rodents and the identification of the exposure levels to which people are exposed.
